# Evaluation of tissue displacement and regional strain in the Achilles tendon using quantitative high-frequency ultrasound

**DOI:** 10.1371/journal.pone.0181364

**Published:** 2017-07-20

**Authors:** Stijn Bogaerts, Catarina De Brito Carvalho, Lennart Scheys, Kaat Desloovere, Jan D’hooge, Frederik Maes, Paul Suetens, Koen Peers

**Affiliations:** 1 Department of Development & Regeneration, KULeuven / Department of Physical Medicine & Rehabilitation, University Hospitals Leuven, Leuven, Belgium; 2 ESAT/PSI & UZ Leuven, MIRC, KULeuven and University Hospitals Leuven, Leuven, Belgium; 3 Department of Development & Regeneration, Institute for Orthopedic Research and Training (IORT), KULeuven / Division of Orthopedics, University Hospitals Leuven, Leuven, Belgium; 4 Clinical Motion Analysis Laboratory, Department of Rehabilitation Sciences, KULeuven and University Hospitals Leuven, Leuven, Belgium; 5 Department of Cardiovascular Sciences, University Hospitals Leuven, Leuven, Belgium; University of Rochester, UNITED STATES

## Abstract

The Achilles tendon has a unique structure-function relationship thanks to its innate hierarchical architecture in combination with the rotational anatomy of the sub-tendons from the triceps surae muscles. Previous research has provided valuable insight in global Achilles tendon mechanics, but limitations with the technique used remain. Furthermore, given the global approach evaluating muscle-tendon junction to insertion, regional differences in tendon mechanical properties might be overlooked. However, recent advancements in the field of ultrasound imaging in combination with speckle tracking have made an intratendinous evaluation possible. This study uses high-frequency ultrasound to allow for quantification of regional tendon deformation. Also, an interactive application was developed to improve clinical applicability. A dynamic ultrasound of both Achilles tendons of ten asymptomatic subjects was taken. The displacement and regional strain in the superficial, middle and deep layer were evaluated during passive elongation and isometric contraction. Building on previous research, results showed that the Achilles tendon displaces non-uniformly with a higher displacement found in the deep layer of the tendon. Adding to this, a non-uniform regional strain behavior was found in the Achilles tendon during passive elongation, with the highest strain in the superficial layer. Further exploration of tendon mechanics will improve the knowledge on etiology of tendinopathy and provide options to optimize existing therapeutic loading programs.

## Introduction

### Structure and function of tendons

Tendons in the human body have a hierarchical structure consisting of collagen triple helices, fibrils, fibers and fascicles [1]. The Achilles tendon (AT) has an extra hierarchical level as it is comprised of the sub-tendons of three muscles of the triceps surae (lateral gastrocnemius, medial gastrocnemius and soleus) [2]. The macrostructure of the AT is even more complex due to its twisted anatomy where fascicles undergo some degree of a counterclockwise (right AT) or clockwise (left AT) rotation, moving from proximal to distal [3]. This complex structure-function relationship leads to a fine balance between resisting tension and allowing compliance [4] as the function of tendons is more complex than just transmitting force from muscle to bone. They store and release energy, protect muscle from stretch damage, allow favorable muscle output and enhance muscle performance [5].

Quantification of the mechanical behavior of the muscle-tendon unit in-vivo during deformation is needed to further elucidate the interplay between structure and function. The most common approach to do this has so far been the tracking of a reference point during an isometric contraction. The most commonly used reference points are the myotendinous junction (of the medial gastrocnemius or soleus) and the calcaneal insertion [6]. This then typically leads to a “global” force-elongation curve, of which the slope is a measure for the stiffness of tendon, relating to the global mechanical properties of the tendon. Force divided by the cross-sectional area of the tendon then leads to stress, elongation divided by the resting length of the tendon on the other hand leads to strain. From this, a stress-strain curve can be derived, of which the slope can be interpreted as Young’s modulus, relating to the intrinsic material properties of the tendon, irrespective of its dimensions [7].

However, quantification of the "global" deformation of myotendinous junction to insertion of the Achilles tendon has its technical limitations [8]. Furthermore, recent studies have shown an important non-uniform deformation pattern in the muscle-tendon unit of the triceps surae, along its length as well as antero-posterior, as could be expected from the rotational anatomy described above [9]. It is known that in the majority of tendons, force transmission occurs mainly along the individual fascicles that can act as independent structures [10]. This intratendinous non-uniform behavior would go undetected when using a global evaluation from myotendinous junction to insertion. In a proximo-distal direction, it has been shown that strain and stiffness levels in the aponeurosis region of the tendon differ from the tendon proper [6]. The bulging of underlying muscles at the aponeuroses level has been suggested as a possible explanation [11]. Even more interesting, at intratendinous level, a cadaver study by Lyman et al. [12], using an invasive technique, has shown a non-uniform strain distribution with the superficial layer of the AT demonstrating a higher strain than the deep layer. Lersch et al. [13] have also shown the influence of calcaneal position on the intratendinous strain distribution, where an eversion led to higher strain in the medial and central distal AT.

Recent evolution in dynamic ultrasound imaging has also led to non-invasive evaluation of these deformation patterns. Arndt et al. [14] were the first to show a larger displacement in the deep layer of the AT during a passive elongation. These findings were confirmed by the group of Slane et al. [15] who later also showed that aging led to a more uniform deformation pattern [16]. Handsfield et al. [5] have recently developed a computational model and found that intratendon sliding and differential muscle force output appear to be the main contributors to this non-uniform deformation pattern. Further knowledge on the biomechanical behavior at intratendinous level is needed to elucidate the role of this non-uniform behavior in pathogenesis of tendinopathy and its place in treatment.

### Ultrasound and speckle tracking

Most of the in-vivo studies mentioned above used ultrasound (US) imaging with speckle tracking approaches to investigate mechanical properties of tendons [14,15]. In summary, an US wave is emitted into the body and the US transducer converts the reflected and scattered US waves into radiofrequency (RF) data. These RF data are afterwards converted to B-mode data and contain information on the internal structure of the scanned tissue and is obtained by coherent summation of signals from scatterers (reflectors), which are typically smaller than the spatial resolution of the imaging system [17]. When this summation occurs, an interference pattern, named speckle, is obtained [18]. Speckles have a granular texture of bright and dark spots. Although classified as noise, speckles are deterministic artifacts, meaning that if the same tissue is evaluated at two different time points, without any changes to the structure of the tissue, the speckle pattern will remain constant. Speckle patterns are correlated with the spatial resolution of the US acquisition system. In other words, if lower spatial resolutions are used, speckle patterns will result from large structures. When using higher spatial resolutions, speckle patterns will result from smaller structures.

Different transducers or acquisition systems can be used to acquire US images of tendons. Depending on the type and properties of the transducer, different spatial resolutions can be obtained. Spatial resolution describes the ability to distinguish between objects located at different positions in space and it is commonly defined in two directions: along the beam propagation direction (axial resolution) and perpendicular to this (lateral resolution). By definition, the axial resolution of the US system corresponds to the capability of the system to distinguish between echoes originated from two objects lying one behind the other. In the case of tendon imaging, it would correspond to discriminate between two fascicles with one lying superficial and one lying deeper. Lateral resolution is then the ability to discriminate between two objects situated side by side. In the case of tendons, it would correspond to discriminate an object lying proximal from one lying more distal.

One of the novelties of this work is that the use of a higher frequency (21 MHz) US acquisition system would enable tracking speckle patterns of smaller structures and hence allow for a description of the inter-fiber and inter-fascicular deformation.

**Fig 1:**
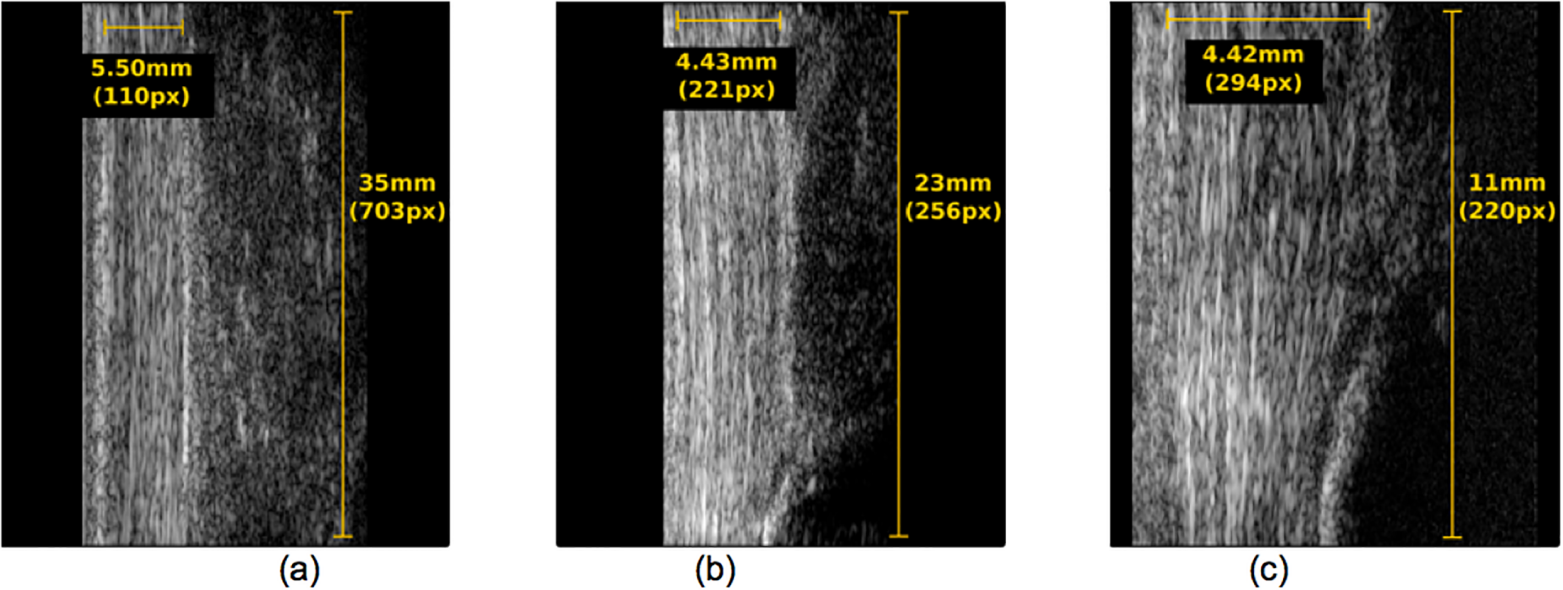
US images of an Achilles tendon acquired with different central frequencies. **(a) 10MHz transducer, (b) 21MHz transducer (c) 40MHz transducer**. Tendon width, length and corresponding image resolution is annotated in yellow.

In order to demonstrate the advantages of such a system, a qualitative comparison was performed (Fig 1) between a conventional 10 MHz US acquisition system (L14-5/38 Linear transducer, Ultrasonix Medical Corporation, Canada) and two transducers of the Vevo2100 (MS250 –central frequency 21 MHz and MS550 –central frequency 40 MHz).

As Fig 1 shows, the AT is represented with more detail in Fig 1(b) and 1(c) than in Fig 1(a). The ultrasound axial resolution of these images is 0.308 mm, 0.0367 mm and 0.0192 mm respectively.

**Fig 2:**
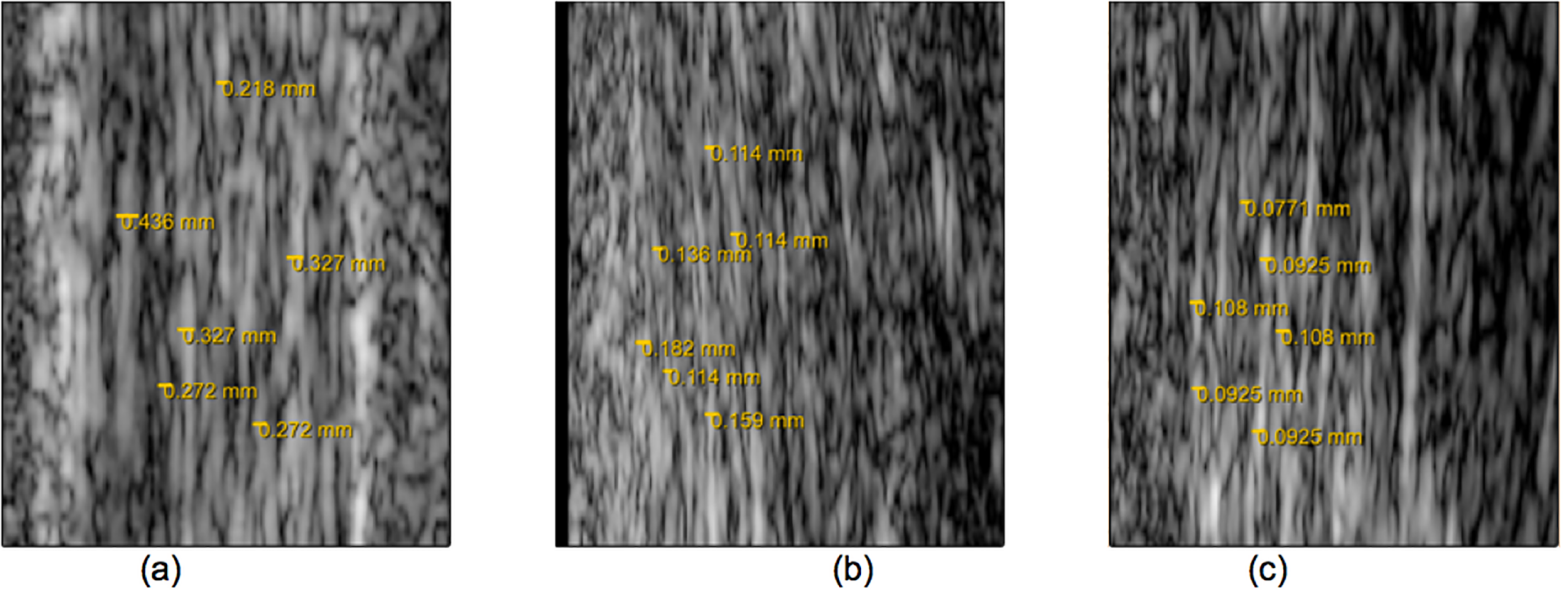
Close-up of Fig 1. Speckle pattern width is measured for the 10MHz image (a), 21MHz image(b) and 40MHz image(c).

A better perspective on the actual resolution of the different US systems is presented in Fig 2, which are close-ups of Fig 1.

As these images demonstrate, with the 10 MHz system it is possible to measure a striated speckle pattern with an average width of +/- 0.30 mm, which would correspond to structures smaller than the system’s axial resolution (0.308 mm), being large fascicles [2]. On the other hand, with the 21 MHz and 40 MHz system, the striated speckle pattern that can be measured has an average width of +/- 0.14 mm and +/- 0.0951 mm, respectively. The speckle pattern obtained from the 21 MHz and 40 MHz transducers are then assumed to represent tendon structures with sizes smaller than the axial resolution of these transducers (0.0367 mm and 0.0182 mm, respectively). This corresponds to tendon fibers [2]. However, this higher resolution along the beam propagation direction is compromised by a reduction of the field of view along the lateral direction (35 mm for 10 MHz, 23 mm for 21 MHz and 11 mm for 40 MHz). The ultrasound lateral resolution of these three images is then 0.039 mm, 0.10 mm and 0.033 mm, respectively.

Considering both lateral and axial resolution, together with the field of view, the US acquisition system used in this work was 21 MHz, which was assumed to allow improved tracking of inter-fiber and inter-fascicle deformation, due to its higher axial resolution (0.0367 mm), while maintaining a reasonable field of view (23 mm). Lateral resolution was not strongly considered because fibers and fascicles have been reported to be elongated structures with longer dimensions [19] and lateral force transmission to be small [10].

From speckle tracking perspective, Heyde et al. [20] compared the performance of two speckle tracking approaches, being block-matching and image registration methods, for cardiac strain estimation. They found that image registration methods yielded slightly better results. Considering also the non-uniform deformation of tendons and the better performance of image registration methods, a non-rigid image registration method was considered the preferred speckle tracking approach.

### Purpose

The objective of this study was to quantify the intratendinous deformation patterns of normal Achilles tendons in-vivo by means of high-frequency ultrasound based speckle tracking. The displacement and regional strain in the superficial, middle and deep layer were evaluated during passive elongation and isometric contraction. Since non-invasive techniques to establish ground-truth are lacking, validation was based on results of previous research in this field. It was hypothesized that highest displacement would be found in the deep layer, as was previously shown by others [14,15]. Quantification of regional strain has so far been even less successful and more challenging [21,22], but based on a cadaver study by Lyman et al. [12], it was hypothesized that the highest regional strain would be found in the superficial layer.

## Methods

### Set-up

The KU UZ Leuven ethics committee approved this specific study and gave it number s57302. After providing written consent, participants filled in a document with demographic questions and completed the VISA-A questionnaire, a validated measure to evaluate tendon health and function [23]. Subjects with VISA-A scores lower then 100, previous history of rupture, surgery, and systemic or neuromuscular diseases were excluded. A convenience sample of subjects was recruited from a group of co-workers at the lab. Participants were asked to refrain from physical activity the day before and the day of the test, other than normal ambulation required in daily life. The participant lay prone on a table, knees extended, with the foot fixated in an isokinetic testing device according to the manufacturer’s guidelines (Biodex system 4 PRO, Biodex Medical Systems, Inc., Shirley, New York). After a standardized warm-up of 5 repetitions of concentric plantar- and dorsiflexion through 20° range of motion, starting from a neutral position, the ultrasound probe was attached to a custom-made holder at the mid-portion of the Achilles tendon. The position of the holder was marked on the skin with a marker to allow reproducible positioning on day 2 for test-retest reliability trials. Two motions were used in randomized order by coin toss between subjects and between days: 2 repetitions of maximal voluntary isometric contraction in a neutral ankle position during 5 seconds, and 2 repetitions of passive elongation from 10° plantarflexion to 10° dorsiflexion and back at 15°/sec. Data from the Biodex machine were collected after finishing all testing procedures. The same procedure was repeated the next day at the same time of the day using the same setup.

### Ultrasound

2-dimensional B-mode US images were acquired during each motion using a high-frequency US system from FujiFilm VisualSonics Inc. (Vevo 2100—Amsterdam, The Netherlands). As mentioned in the introduction, a transducer (MS 250) with a central frequency of 21MHz was used and the acquired images had a spatial image resolution of 0.02x0.09mm. A dynamic sequence of 2D images was acquired during 5 seconds with a temporal resolution of 100 frames per second.

#### Tissue displacement and strain estimation

As mentioned in the introduction, a non-rigid image registration method was the preferred speckle tracking method due to its ability to recover non-uniform deformation. Tissue displacement was then estimated by finding the transformation *T(x)* that maximized the spatial correspondences when deforming a moving image *(I*_*M*_*)* to match a fixed image *(I*_*F*_*)*. These correspondences are maximized by optimizing a cost function *(C)*, defined according to a similarity measure *(S)* and a penalty term *(P)*, with respect to *T(x)* as presented in Eq 1.

$$\begin{array}{ll} T\left(x\right) & =arg\underset{T\left(x\right)}{min}C\left(T\left(x\right);I_{F},I_{M}\right)\\ C\left(T\left(x\right);I_{F},I_{M}\right) & =\;-S\left(T\left(x\right);I_{F},I_{M}\right)+\;\;\gamma P\left(T\left(x\right)\right) \end{array}$$ (1)

A cubic Bspline transformation T was used in this work. In this type of transformation, the control points, placed in a uniform grid, are displaced to deform the moving image to match the fixed image. The spacing between the control points defines the locality of the transformation: the smaller the spacing, the more local deformations are obtained. The displacement of the pixels at non-control positions is obtained by cubic interpolation. Sum of squared differences between intensity values of corresponding locations in both images was used as similarity measure and a bending energy penalty P with a weight *γ* of 0.5 was used [24]. This penalty term penalizes sharp discontinuities in the transformation. A three-level multi-resolution strategy using a pyramidal scheme was used with a grid spacing of 4, 2 and 1 pixels from coarser to finer resolution. As optimizer, a quasi-Newton limited memory BFGS (Broyden—Fletcher—Goldfarb—Shanno) [25] was employed to minimize the cost function *C* because of its speed and memory efficiency [20].

After all trials were visually reviewed to ensure that there were no artifacts (e.g. probe release, air bubble) and a stable speckle pattern was visualized, a region of interest (ROI) containing only tendon material was selected, to limit the calculation of deformation to the tissue of interest. In order to reduce computational effort, an interval of 100 frames, out of 500 frames of the cine-loop, was selected.

A pair-wise registration of consecutive frames in the sequence was favored due to the high speckle correlation between images acquired with a low temporal difference. Once every consecutive pair of images was registered, the transformation between the last frame of the cine-loop (100^th^) and the first one was obtained by composition of the intermediate pair-wise image transformations. At the end, point-wise displacement maps were obtained along the major deformation direction (Δ*d*_*major*_) representing the tissue displacement in the longitudinal direction (i.e. the principal strain direction), relative to the starting position in the first frame. This entire framework was implemented using elastix [26,27].

**Fig 3:**
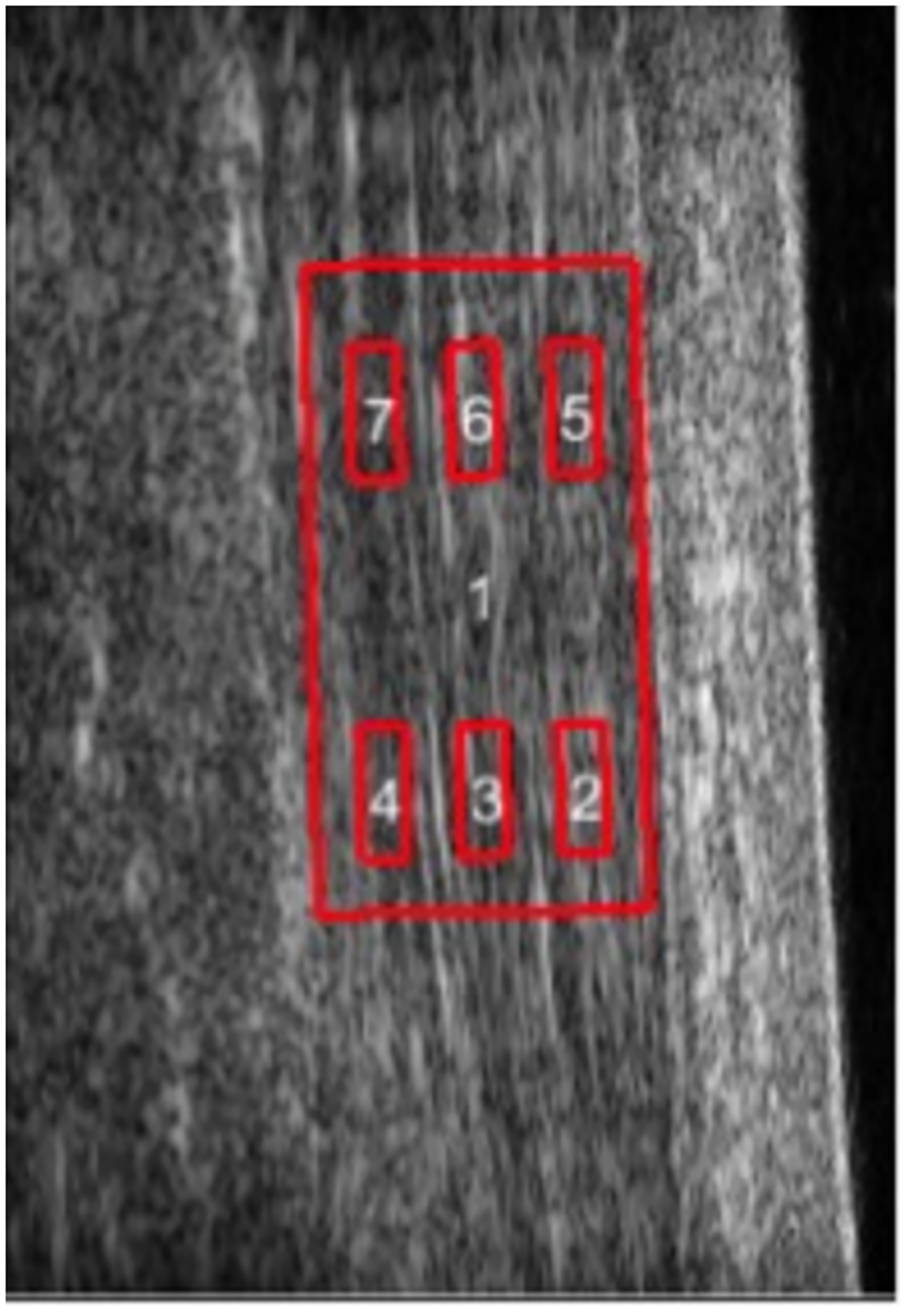
2D US image of volunteer with selected regions of interest (1) and subregions (2–7) delimited in red.

The ROI selected at the pre-processing step was re-used to automatically define 6 sub-regions (3 proximal and 3 distal), consisting of a deep, middle and superficially located sub-region as represented in Fig 3. The deep, middle and superficial sub-regions were automatically placed at 25, 50 and 75% of the selected global ROI width, and the proximal and distal at 25 and 75% of the global ROI length. The deep-middle-superficial sub-division was based on clinical reasoning where it was expected that the three muscles of the triceps surae account for three layers of the sub-tendons (lateral gastrocnemius, medial gastrocnemius and soleus) in the Achilles tendon. The proximal versus distal sub-division was done to include the potential to evaluate possible longitudinal differences.

The average displacement within each sub-region along the major deformation direction $\bar{\Delta d_{major}}$ was computed directly from the point-wise displacement maps. Regional strain (Eq 2) was computed along the major deformation direction between two different regions (Ri and Rj). Superficial strain was estimated between R5 and R2 (Ri = R5 and Rj = R2, Eq 2), medial strain was computed between R3 and R6 (Ri = R3 and Rj = R6, Eq 2) and deep strain was computed using R7 and R4 (Ri = R7 and Rj = R4, Eq 2). *L*_*initial*_ represents the distance between the central position of Ri and Rj.

$$RegionalStrain=\frac{\bar{\Delta d_{major}R\text{i}}-\bar{\Delta d_{major}R\text{j}}}{L_{initial}}\times 100$$ (2)

### Statistics

Statistical analysis was performed using SPSS (IBM, New York, NY, USA). Test-retest reliability (intraday: between 2 repetitions on the same day—interday: between average of 2 repetitions on 2 consecutive days) was evaluated for results of displacement in the superficial layer during passive trials in the right leg of all 10 subjects. Results were assessed using intraclass correlation coefficients (ICC). Standard error of measurement (SEM) was then derived from ICC’s.

After confirming normal distribution of results with Shapiro-Wilk’s test, non-uniform deformation of the different layers was evaluated using a two-sided paired t-test with alpha-level set at 0.05. A comparison between the superficial, middle and deep layer during passive as well as isometric trials was made. Also, the comparison between the absolute difference in mean displacement between layers (superficial to deep) during passive versus isometric trials was made.

## Results

10 asymptomatic subjects (6 male– 4 female; 26 (SD 3) years; 72 (SD 14) kg; VISA-A 100 (SD 0) %) participated in this experiment with both Achilles tendons tested, leading to a total of 20 tendons investigated. The mean torque, relative to body weight, generated on the Biodex was 79 N/kg (SD 32 N/kg) during the isometric trials and 7,5 N/kg (SD 3,4 N/kg) during the passive trials. The full data set is available in the supporting information (S1 Table).

### Test-retest reliability

ICC was 0.86 for intraday and 0.72 for interday measurements, leading to a SEM of 0.35 mm and 0.44 mm respectively.

### Tissue displacement

**Fig 4:**
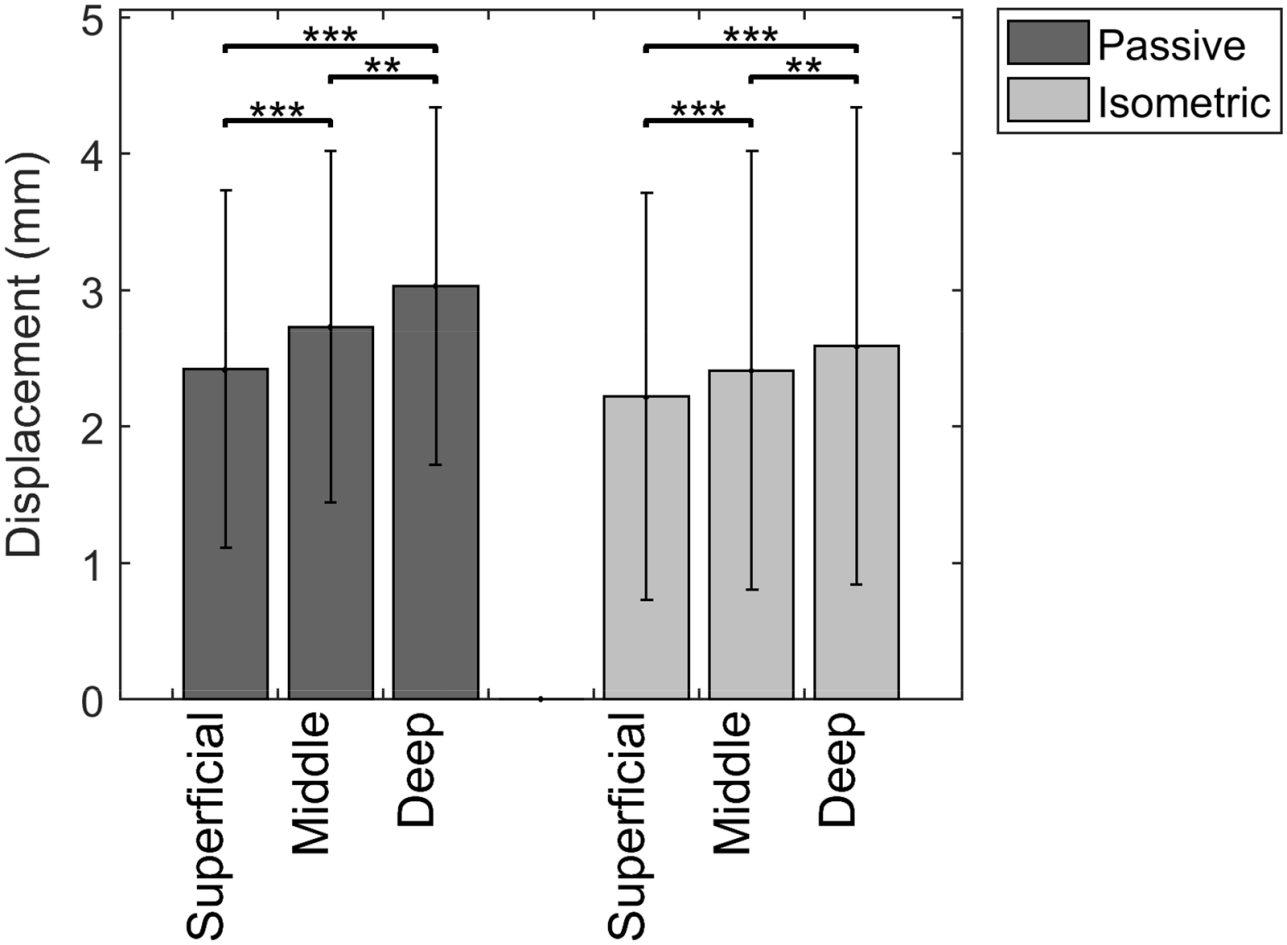
Non-uniform displacement along the major deformation direction (*** = p < 0.001).

There was a significantly different tissue displacement when comparing the three layers during passive as well as isometric trials (p < 0.001). The deep layer of the tendon moved most with an average displacement of 3,03 mm during passive elongation and 2,59 mm during isometric contraction (Fig 4).

### Regional strain

**Fig 5:**
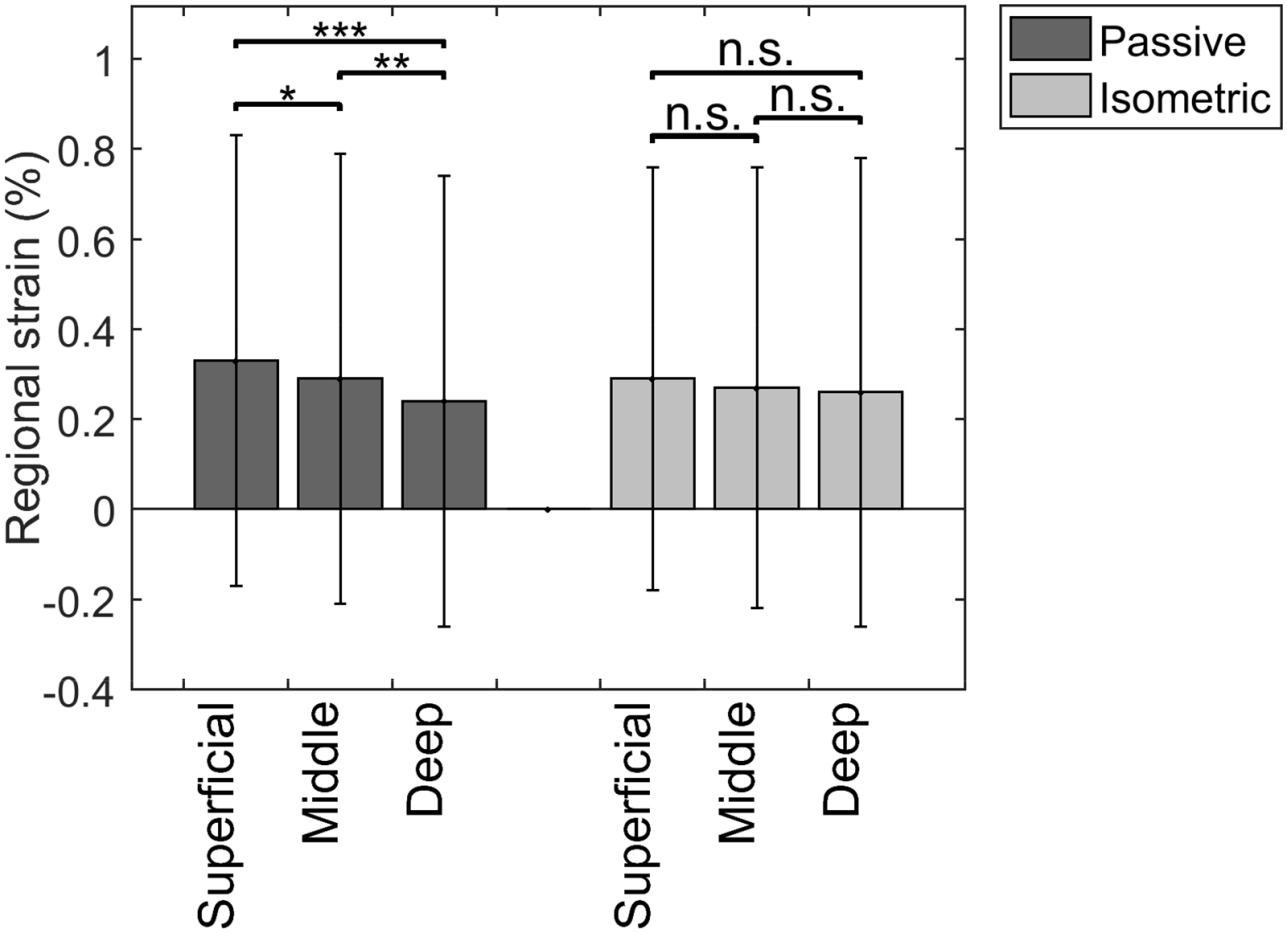
Non-uniform regional strain along the major deformation direction (** = p = 0.002 / *** = p < 0.001 / n.s. = non significant).

There was a significantly different regional strain when comparing the three layers, but only during passive elongation. The non-uniform regional strain was not significantly different during the isometric trials. Highest strain was found in the superficial layer with an average of 0,33% during passive elongation and 0,29% during isometric contraction (Fig 5).

### Relative displacement

There is an absolute difference in displacement of 0,34 (SD 0,24) mm between the superficial and middle layer and of 0,34 (SD 0,23) mm between the middle and deep layer during passive elongation. For isometric contraction, the absolute differences in displacement are 0,25 (SD 0,24) mm for superficial-middle and 0,24 (SD 0,23) mm for middle-deep.

**Fig 6:**
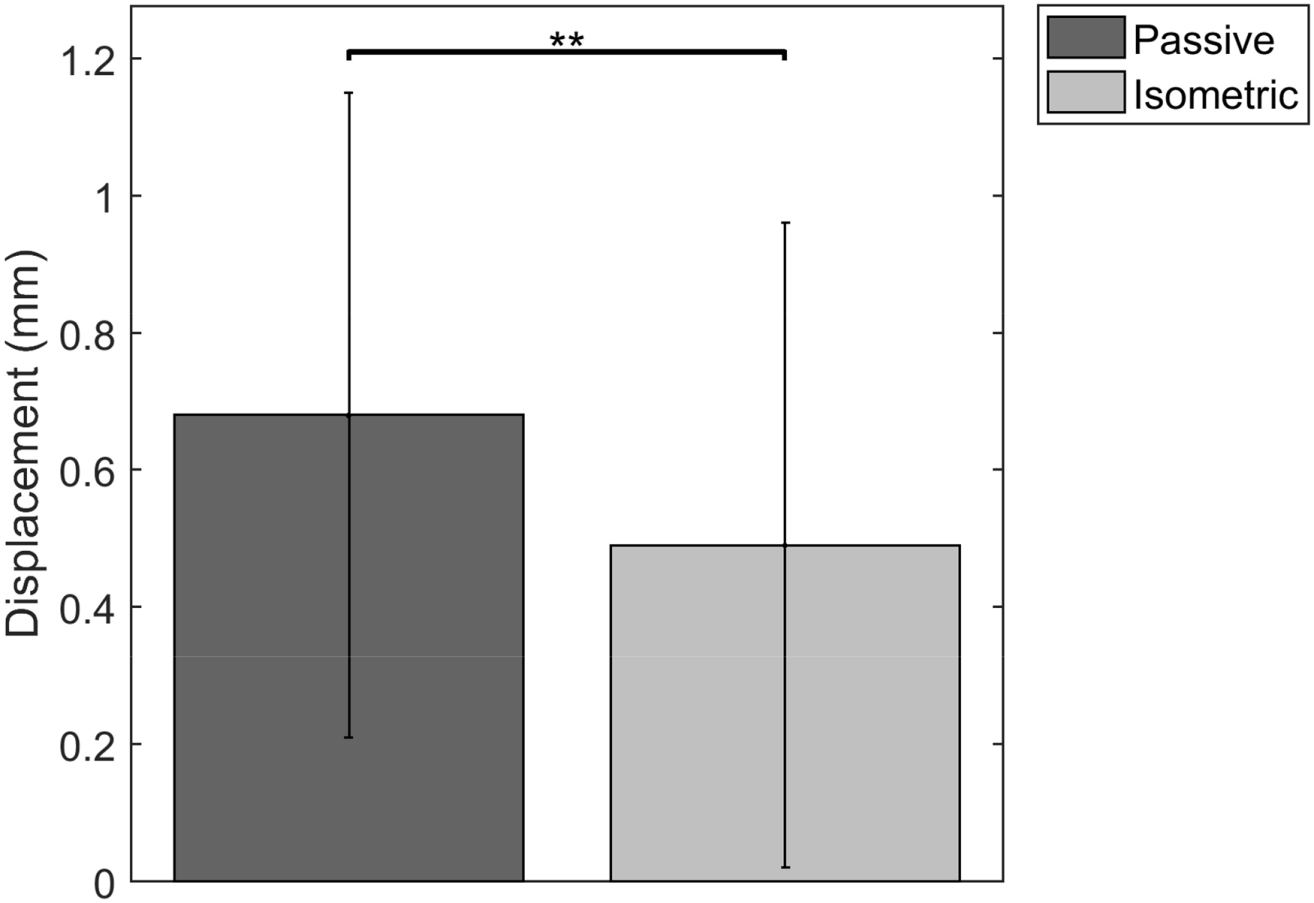
Absolute difference in mean displacement of superficial versus deep layer (** = p = 0,002).

Looking at absolute difference in mean displacement between the superficial and deep layer there is a significantly larger difference during the passive elongation, compared to the isometric deformation (Fig 6).

## Discussion

The objective of this study was to quantify the intratendinous deformation patterns of normal Achilles tendons in-vivo by means of high-frequency ultrasound based speckle tracking. Results showed that the Achilles tendon displaces non-uniformly with a higher displacement found in the deep layer of the tendon. This is in line with the findings of Slane et al. and Arndt et al. [14,15]. Adding to this, this study showed, using a non-invasive method, a non-uniform regional strain behavior in the Achilles tendon during passive elongation, with the highest strain found in the superficial layer. There was a similar trend observed during isometric contraction, however not significant. This is in line with the cadaver work of Lyman et al. [12] and in-vivo work by Chimenti et al. on the insertional Achilles tendon [28]. Previous cadaver studies using invasive techniques on patellar tendons [29] and supraspinatus tendons [30] also showed that joint-sided strain was lowest, which would relate to the superficial strain the Achilles tendon being highest.

A few possible reasons for the non-uniform behavior in Achilles tendons have been recently described [31]. The rotational anatomy of the Achilles tendon [3] provides a basis for non-uniform deformation between layers. At the insertional level of the Achilles tendon, the superficial fibers relate mostly to the gastrocnemius medialis subtendon and the deep fibers mostly to the gastrocnemius lateralis subtendon [9]. In their anatomical study, Pekala et al. [9] hypothesize that the deep layer of the AT in general twists more than the superficial. This could lead to a higher pre-tension in the deep part of the tendon, leaving little to no margin for extra straining during deformation and therefore more displacement in this layer of the tendon. For the same level of force going through the whole tendon, the superficial part on the other hand could then still undergo more straining during deformation. Besides differences in morphology and neuromuscular activation patterns in the triceps surae, there might also be local material differences at the level of the tendon [31]. This was already described in the patellar tendon, where tendon fascicles from the anterior portion of the human patellar tendon in young men displayed considerably greater peak and yield stress and tangent modulus compared with the posterior portion of the tendon, indicating region-specific material properties [32,33].

The difference in absolute difference in mean displacement between passive and isometric trials (Fig 6) falls in line with previous research from Finni et al. [34]. They have described the influence of active versus passive muscle contribution to the behavior of muscle shear, which is believed to have an influence on tendon behavior. During passive conditions, there is a slack and compliant connection between muscle bellies of the triceps surae, leaving margin for an independent non-uniform behavior. During active conditions, the tensing of muscle connections might lead to more uniform behavior.

As described in the introduction, since we are evaluating at fascicle and fiber level, the non-uniform behavior described in this paper supposedly links to sliding at intratendinous level. This sliding might even be more important than pure tensile—longitudinal strain, as Thorpe et al. [35] nicely summarize ex vivo and in vitro research, stating that “… at low force, there is sliding between fibers/fibril, but no real fiber extension. At higher force there is interfascicular matrix sliding”. It has also been shown that changes at the interfascicular matrix is an important factor to consider in the pathogenesis of tendinopathy [36]. With respect to pathogenesis and the effects of ageing and loading, other research has shown that ageing leads to changes in the tendon morphology and mechanics [37] with increase in non-enzymatic crosslinks and as a consequence an expected increase in stiffness [38]. However, stiffness seems to decrease as we age [39]. This might be due to the loss of interfascicular sliding because of non-enzymatic crosslinks, turning fascicles more vulnerable to local tensile strain, which can consequently lead to damage. This local damage and loss of local tensile stiffness from the fascicles could then be the tipping point where the decline of stiffness starts as seen in elderly, but also leading to stress-shielding [40] and development into pathologic tendons [41]. The possibility to evaluate in-vivo tendon sliding could therefore be a valuable tool to detect changes in tendon function before onset of structural pathology or symptoms.

The absolute values of tendon displacement in the data presented here are smaller when compared with previous research applying similar techniques. Arndt et al. [14] reported displacements ranging from 4,6 to 13,5 mm. The results from Slane et al. [15] ranged between 4 and 8 mm. Results in the current study showed displacements only up to 2,95 mm. This could be explained by the differences between studies in range and speed of motion, and position of measurement along the free tendon. The study of Arndt et al. used a similar set-up to this study, but with a larger range of motion, going from 20° plantarflexion up to 15° dorsiflexion at an angular velocity of 15°/sec. The study of Slane et al was slightly different with a position from 0° to 30° plantarflexion at a rate of 0,5 Hz (correlating with a peak angular speed of 90°/sec at mid range of motion), much higher then 15°/sec as used in this study. The Achilles tendon can be considered a visco-elastic tissue, where at low loading rates and slower loading the viscous behavior more important with high energy absorption, leaving more time for creep phenomena and higher local shear strain [42,43] and so conversely lower displacement values. This means that in the set-up used, with a low loading rate (15°/sec) during the passive trials, the tendon will have enough time to deform and creep.

The absolute values of strain in our study are small and much less than values that have been previously reported in the literature documenting global in-vivo strain measurement values from 2 up to 11% [6]. However, these small values are in line with results from in vitro research and cadaver studies at intratendinous level. Screen et al. [44] showed, using microscopic imaging-based analysis that local strains at fascicle level are smaller than globally applied strains and never exceeded 1.2%, even at 8% gross applied strain. A study by Arnockzy et al. [45] on rat tails reported similar findings. The regional strain values in this current study are nicely in line with the intratendinous strain measured on cadavers by Lyman et al. [12] where values ranged from 0,07% to 1,11% in different areas of the insertional Achilles tendon. The only other study evaluating tensile strain in-vivo, by Chimenti et al. [28], used radiofrequency data from an ultrasound device of lower resolution (10 MHz). Importantly, the set-up was different, as subjects in those experiments were standing or positioned in partial squat during the ankle dorsiflexion motion. Only results from the standing position trials can be compared to our results, given the fact that subjects in our study had their leg extended during all trials (passive and isometric). Strain results in the standing trials by Chimenti et al. [28] varied around 3.33% for the deep and 3.57% for the superficial region. It could be expected that strains are higher during a weight-bearing exercise when compared with a passive elongation or isometric contraction. An important limitation in their results is the high degree of variability, as was also the case in our results.

A limitation of this study is the small sample size with only 10 subjects (20 tendons) evaluated. As was previously mentioned in other research [46], the range of mechanical parameters in healthy controls varies based on many factors (external, e.g. activity, and internal, e.g. material properties). The ICC values were moderate for interday to good for intraday analysis. A second limitation is the high variability in strain values. It was stated previously by other authors that a high variability in strain estimation remains when using commercially available speckle tracking techniques [22]. It is beyond the scope of this article to go in depth on these technical details. However, it is reasonable to believe that a certain degree of variability in local strain results, when measured using ultrasound based speckle tracking, is physiological and not only due to technical limitations or noise. The high amount of interindividual variation in degree of rotation as seen by anatomical studies [3,9] could already partly explain the high interindividual variation in strain and deformation patterns. An important technical consideration is the fact that tracking of tendon motion with 2D-ultrasound is confronted with out-of-plane motion, which is always to be expected due to the complex 3D-deformation of tendons. However, the impact of this is limited thanks to the high frame-rate of 100 frames per second that is used in these experiments. It has been shown that small frame-to-frame displacements increased the potential to keep scatterers in view, which decreases the effect of out-of-plane motion and therefore enhances the ability to accurately track motion [47]. Also, as mentioned above, all trials were visually reviewed to ensure there were no artifacts (e.g. probe release, air bubble) and a stable speckle pattern was visualized. In the future, 3D-ultrasound will most likely become a tool to overcome the problem of out-of-plane motion artifacts.

**Fig 7:**
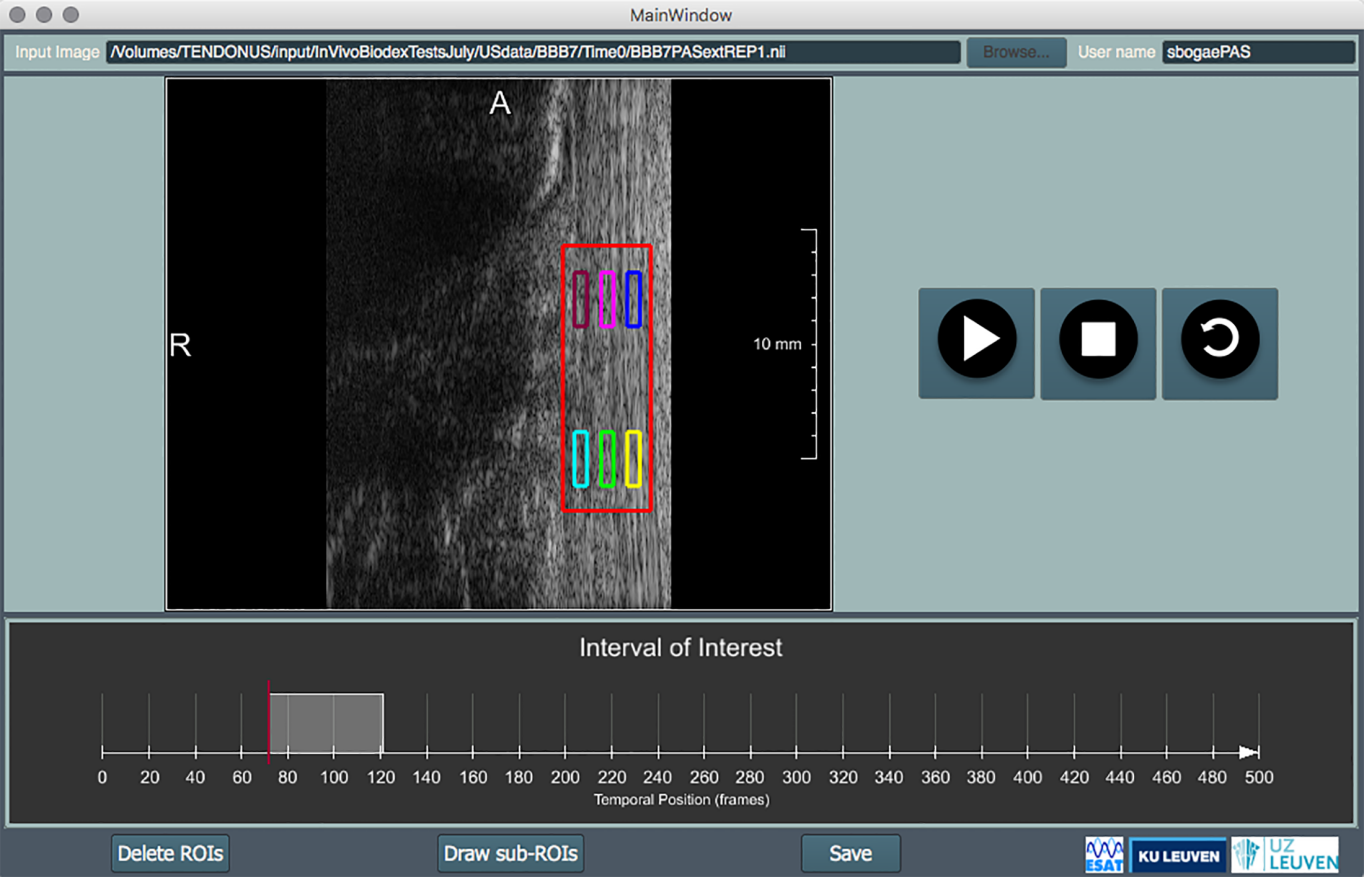
Interactive application.

To improve clinical applicability, this study also developed an interactive application (Fig 7) to provide researcher SB with an adequate environment to segment and select the ROI’s and time-intervals. This could potentially provide clinicians with a tool to track intra-individual change over time. Still, attention has to be made to use a reproducible set-up with adequate image acquisition and good control of image quality. The definition of population based cut-off values will be much harder, given the previously stated methodological and physiological between-subject variability.

Future research should include pathological tendons in different stages of symptomatology, pathology and age, since only a few studies have previously investigated the mechanical deformation in degenerated tendons with computational models [48] or other techniques [46,49,50]. Also, compressive or axial strain [51] is an interesting way of further investigating the local mechanical behavior of healthy and pathologic tendons. Another possible addition could be the combined evaluation of global and regional strain with parallel tracking of the musculotendinous junction and insertion on the calcaneus and torque evaluation. This would provide further insight in the complex structure-function relationship of the triceps surae muscle-tendon unit.

## Conclusions

The objective of this study was to quantify the intratendinous deformation patterns of normal Achilles tendons in-vivo by means of ultrasound based speckle tracking. The first novelty of this work was the use of a high-frequency (21 MHz) ultrasound acquisition system. This allowed the tracking of speckle patterns of smaller structures and henceforth a better description of the inter-fiber and inter-fascicular deformation. Secondly, an interactive application was used to improve clinical applicability. The displacement and regional strain in the superficial, middle and deep layer were evaluated during passive elongation and isometric contraction. Building on previous research, results showed that the Achilles tendon displaces non-uniformly with a higher displacement found in the deep layer of the tendon. Adding to this, a non-uniform regional strain behavior was found in the Achilles tendon during passive elongation, with the highest strain in the superficial layer.

## Supporting information

S1 Table Full data set. (PDF)
