# Supplementary material for: Evaluation of tissue displacement and regional strain in the Achilles tendon using quantitative high-frequency ultrasound
Source: PLoS One. 2017 Jul 20;12(7):e0181364. doi: 10.1371/journal.pone.0181364 (PMC5519157; doi:10.1371/journal.pone.0181364)
Supplement: S1 Table — (PDF) [file pone.0181364.s001.pdf]

Patiënt	Gender	Age	Weight	Leg	Activation	REP
1	Female	25	59	L	PAS	1
1	Female	25	59	L	PAS	1
1	Female	25	59	L	PAS	2
1	Female	25	59	L	PAS	2
1	Female	25	59	L	ISO	1
1	Female	25	59	L	ISO	1
1	Female	25	59	L	ISO	2
1	Female	25	59	L	ISO	2
1	Female	25	59	R	PAS	1
1	Female	25	59	R	PAS	1
1	Female	25	59	R	PAS	2
1	Female	25	59	R	PAS	2
1	Female	25	59	R	ISO	1
1	Female	25	59	R	ISO	1
1	Female	25	59	R	ISO	2
1	Female	25	59	R	ISO	2
2	Male	27	78	L	PAS	1
2	Male	27	78	L	PAS	1
2	Male	27	78	L	PAS	2
2	Male	27	78	L	PAS	2
2	Male	27	78	L	ISO	1
2	Male	27	78	L	ISO	1
2	Male	27	78	L	ISO	2
2	Male	27	78	L	ISO	2
2	Male	27	78	R	PAS	1
2	Male	27	78	R	PAS	1
2	Male	27	78	R	PAS	2
2	Male	27	78	R	PAS	2
2	Male	27	78	R	ISO	1
2	Male	27	78	R	ISO	1
2	Male	27	78	R	ISO	2
2	Male	27	78	R	ISO	2
3	Male	26	62	L	PAS	1
3	Male	26	62	L	PAS	1
3	Male	26	62	L	PAS	2
3	Male	26	62	L	PAS	2
3	Male	26	62	L	ISO	1
3	Male	26	62	L	ISO	1
3	Male	26	62	L	ISO	2
3	Male	26	62	L	ISO	2
3	Male	26	62	R	PAS	1
3	Male	26	62	R	PAS	1
3	Male	26	62	R	PAS	2

3	Male	26	62	R	PAS	2
3	Male	26	62	R	ISO	1
3	Male	26	62	R	ISO	1
3	Male	26	62	R	ISO	2
3	Male	26	62	R	ISO	2
4	Male	33	102	L	PAS	1
4	Male	33	102	L	PAS	1
4	Male	33	102	L	PAS	2
4	Male	33	102	L	PAS	2
4	Male	33	102	L	ISO	1
4	Male	33	102	L	ISO	1
4	Male	33	102	L	ISO	2
4	Male	33	102	L	ISO	2
4	Male	33	102	R	PAS	1
4	Male	33	102	R	PAS	1
4	Male	33	102	R	PAS	2
4	Male	33	102	R	PAS	2
4	Male	33	102	R	ISO	1
4	Male	33	102	R	ISO	1
4	Male	33	102	R	ISO	2
4	Male	33	102	R	ISO	2
5	Female	24	64	L	PAS	1
5	Female	24	64	L	PAS	1
5	Female	24	64	L	PAS	2
5	Female	24	64	L	PAS	2
5	Female	24	64	L	ISO	1
5	Female	24	64	L	ISO	1
5	Female	24	64	L	ISO	2
5	Female	24	64	L	ISO	2
5	Female	24	64	R	PAS	1
5	Female	24	64	R	PAS	1
5	Female	24	64	R	PAS	2
5	Female	24	64	R	PAS	2
5	Female	24	64	R	ISO	1
5	Female	24	64	R	ISO	1
5	Female	24	64	R	ISO	2
5	Female	24	64	R	ISO	2
6	Female	23	64	L	PAS	1
6	Female	23	64	L	PAS	1
6	Female	23	64	L	PAS	2
6	Female	23	64	L	PAS	2
6	Female	23	64	L	ISO	1
6	Female	23	64	L	ISO	1
6	Female	23	64	L	ISO	2
6	Female	23	64	L	ISO	2

6	Female	23	64	R	PAS	1
6	Female	23	64	R	PAS	1
6	Female	23	64	R	PAS	2
6	Female	23	64	R	PAS	2
6	Female	23	64	R	ISO	1
6	Female	23	64	R	ISO	1
6	Female	23	64	R	ISO	2
6	Female	23	64	R	ISO	2
7	Male	28	64	L	PAS	1
7	Male	28	64	L	PAS	1
7	Male	28	64	L	PAS	2
7	Male	28	64	L	PAS	2
7	Male	28	64	L	ISO	1
7	Male	28	64	L	ISO	1
7	Male	28	64	L	ISO	2
7	Male	28	64	L	ISO	2
7	Male	28	64	R	PAS	1
7	Male	28	64	R	PAS	1
7	Male	28	64	R	PAS	2
7	Male	28	64	R	PAS	2
7	Male	28	64	R	ISO	1
7	Male	28	64	R	ISO	1
7	Male	28	64	R	ISO	2
7	Male	28	64	R	ISO	2
8	Male	22	86	L	PAS	1
8	Male	22	86	L	PAS	1
8	Male	22	86	L	PAS	2
8	Male	22	86	L	PAS	2
8	Male	22	86	L	ISO	1
8	Male	22	86	L	ISO	1
8	Male	22	86	L	ISO	2
8	Male	22	86	L	ISO	2
8	Male	22	86	R	PAS	1
8	Male	22	86	R	PAS	1
8	Male	22	86	R	PAS	2
8	Male	22	86	R	PAS	2
8	Male	22	86	R	ISO	1
8	Male	22	86	R	ISO	1
8	Male	22	86	R	ISO	2
8	Male	22	86	R	ISO	2
9	Female	26	59	L	PAS	1
9	Female	26	59	L	PAS	1
9	Female	26	59	L	PAS	2
9	Female	26	59	L	PAS	2
9	Female	26	59	L	ISO	1

9	Female	26	59	L	ISO	1
9	Female	26	59	L	ISO	2
9	Female	26	59	L	ISO	2
9	Female	26	59	R	PAS	1
9	Female	26	59	R	PAS	1
9	Female	26	59	R	PAS	2
9	Female	26	59	R	PAS	2
9	Female	26	59	R	ISO	1
9	Female	26	59	R	ISO	1
9	Female	26	59	R	ISO	2
9	Female	26	59	R	ISO	2
10	Male	21	77	L	PAS	1
10	Male	21	77	L	PAS	1
10	Male	21	77	L	PAS	2
10	Male	21	77	L	PAS	2
10	Male	21	77	L	ISO	1
10	Male	21	77	L	ISO	1
10	Male	21	77	L	ISO	2
10	Male	21	77	L	ISO	2
10	Male	21	77	R	PAS	1
10	Male	21	77	R	PAS	1
10	Male	21	77	R	PAS	2
10	Male	21	77	R	PAS	2
10	Male	21	77	R	ISO	1
10	Male	21	77	R	ISO	1
10	Male	21	77	R	ISO	2
10	Male	21	77	R	ISO	2

Time	Displacement (mm)			Strain (%)		
	Superficial	Middle	Deep	Superficial	Middle	Deep
0	-7,6798	-7,6798	-7,6779	-0,6576	-0,6721	-0,6834
1	-5,7399	-5,9848	-6,1084	-1,7735	-1,7058	-1,6043
0	-7,6517	-7,6661	-7,6782	-0,7807	-0,7986	-0,8131
1	-5,1127	-5,6365	-6,1362	-2,1804	-2,1596	-2,0408
0	0,83	0,8294	0,8287	0,0466	0,0478	0,0492
1	1,6477	1,7341	1,7984	0,1285	0,0972	0,062
0	0,6945	0,6011	0,5111	0,091	-0,0022	-0,0861
1	4,8229	5,0761	5,2934	0,4394	0,3528	0,2808
0	-3,7657	-3,6573	-3,5382	-0,1088	-0,093	-0,0747
1	-5,0055	-6,0441	-6,9416	-1,1889	-0,5131	-0,0452
0	-3,724	-3,6236	-3,5142	-0,1103	-0,096	-0,0857
1	-2,4416	-3,2745	-4,0771	-0,6776	-0,5829	-0,4943
0	0,5648	0,4988	0,4316	-0,0197	-0,0125	-0,0038
1	2,3497	2,4638	2,5681	0,0182	0,0178	-0,0001
0	0,5242	0,5826	0,6321	0,0064	0,009	0,0166
1	0,2578	0,2439	0,2288	-0,0349	-0,0363	-0,0384
0	-1,963	-2,6041	-3,2828	0,2599	0,4373	0,6676
1	-2,2921	-2,8042	-3,3402	-0,5896	-0,6092	-0,5808
0	-2,1812	-2,996	-3,8656	0,6595	0,7497	0,9187
1	-1,923	-2,2425	-2,5873	-0,4638	-0,4644	-0,4474
0	1,6653	1,3521	1,0302	-0,1532	-0,2701	-0,3691
1	2,3086	2,5039	2,7143	-0,1072	-0,072	-0,0383
0	0,7317	0,5384	0,3554	0,0119	-0,0949	-0,1796
1	1,3114	1,4484	1,5831	0,2789	0,3338	0,3785
0	-2,4658	-2,8061	-3,1345	0,1386	0,1549	0,1819
1	-1,3766	-1,4999	-1,5943	-0,4984	-0,3638	-0,2221
0	-2,9649	-3,3322	-3,686	0,2109	0,2077	0,2142
1	-2,1061	-2,0546	-1,9599	-0,772	-0,6334	-0,4739
0	0,6496	0,8527	1,0224	0,1266	0,0142	-0,0952
1	0,2526	0,1637	0,0713	0,0276	0,0285	0,0254
0	0,3409	0,3801	0,4134	0,1087	0,0448	-0,0127
1	0,2729	0,3655	0,4655	-0,1123	-0,1198	-0,1346
0	-2,4304	-2,5636	-2,688	-0,5015	-0,4739	-0,4328
1	-1,4753	-2,0895	-2,6793	-0,413	-0,295	-0,153
0	-2,1453	-2,3591	-2,5538	-0,3491	-0,3911	-0,4064
1	-1,4274	-2,005	-2,5588	-0,2252	-0,1447	-0,0369
0	1,3896	1,5519	1,6883	0,0272	0,0832	0,1318
1	3,0933	3,6148	4,0997	1,4842	1,5161	1,5527
0	2,7285	3,2886	3,842	0,376	0,2681	0,1836
1	2,859	3,6118	4,3232	1,5159	1,522	1,5678
0	-1,8717	-2,1437	-2,4327	-0,6184	-0,6986	-0,7726
1	-2,3013	-2,9302	-3,5721	-0,597	-0,409	-0,162
0	-2,2081	-2,4805	-2,7949	-0,4517	-0,528	-0,6007

1	-1,7802	-2,2032	-2,6381	-0,6566	-0,5205	-0,3554
0	2,7953	2,9983	3,2056	0,4907	0,4883	0,4619
1	6,2648	6,6483	7,0218	2,4127	2,4808	2,5519
0	3,8803	4,1068	4,3318	0,493	0,5112	0,5229
1	4,9875	5,1119	5,2406	0,2836	0,2806	0,2819
0	-3,7196	-3,6601	-3,5717	-0,2058	-0,2314	-0,2449
1	-2,7972	-3,136	-3,4845	-0,2713	-0,2386	-0,179
0	-3,6696	-3,6754	-3,6477	-0,7434	-0,831	-0,8914
1	-2,9016	-3,2216	-3,5451	-0,3202	-0,2847	-0,2235
0	2,5506	2,3905	2,2215	0,3033	0,3579	0,4094
1	2,0976	2,3669	2,6451	0,213	0,1715	0,1291
0	2,6613	2,4338	2,2014	0,4026	0,4936	0,5709
1	2,8448	3,2399	3,6587	0,1817	0,1837	0,2082
0	-2,1394	-2,2716	-2,4052	0,7881	0,8653	0,9376
1	-1,7557	-2,1068	-2,4715	-0,7615	-0,7214	-0,6577
0	-2,3228	-2,439	-2,555	0,859	0,9347	1,0038
1	-2,3112	-2,7301	-3,1599	-0,9168	-0,8242	-0,6878
0	3,6362	3,7653	3,9015	-0,8785	-1,1371	-1,3967
1	4,3168	4,6189	4,9601	-0,0597	-0,122	-0,2035
0	1,7748	1,7798	1,787	-0,0948	-0,0779	-0,0623
1	4,0472	4,2412	4,4267	0,3756	0,3433	0,2997
0	-1,1124	-1,4632	-1,8039	-0,1214	-0,1241	-0,1105
1	-2,6441	-3,3236	-3,9887	0,3397	0,3343	0,3965
0	-1,5575	-2,1641	-2,7501	0,159	0,3916	0,653
1	-2,0957	-2,9289	-3,7483	-0,0226	-0,0741	-0,0505
0	0,9342	1,0129	1,0932	-0,2582	-0,2679	-0,265
1	1,1124	1,3061	1,4992	-0,107	0,0441	0,1988
0	0,8516	1,0832	1,3354	-0,2143	-0,2064	-0,1816
1	0,6173	0,8167	1,0191	-0,0303	0,0362	0,1123
0	-1,63	-2,4116	-3,2069	-0,4919	-0,7043	-0,8105
1	-1,8308	-2,2975	-2,7751	-0,4991	-0,5083	-0,4794
0	-1,38	-2,1553	-2,9187	-0,6264	-0,7678	-0,8397
1	-1,7001	-2,1401	-2,5658	-0,2366	-0,2384	-0,2246
0	0,4024	0,551	0,6974	0,2822	0,2955	0,3083
1	1,8551	2,7988	3,7024	0,781	0,7413	0,7712
0	0,6793	0,9837	1,2703	0,36	0,3773	0,3802
1	1,1895	1,9069	2,5804	0,3874	0,3219	0,3169
0	-2,1065	-2,2444	-2,3817	-0,5686	-0,5868	-0,5971
1	-2,503	-2,9329	-3,3463	-0,7356	-0,7049	-0,6537
0	-3,5638	-3,6521	-3,7407	-0,8527	-0,93	-0,9894
1	-2,0662	-2,3897	-2,7039	-0,6165	-0,5926	-0,5521
0	3,4206	2,9637	2,4942	0,4683	0,7578	1,0306
1	3,0331	3,0521	3,0658	0,3326	0,3286	0,3221
0	3,2029	2,8204	2,4561	0,8313	0,8209	0,8119
1	4,4063	4,6427	4,8675	-0,086	-0,01	0,0723

0	-2,6227	-2,3035	-1,9587	0,3034	0,2509	0,1988
1	-3,0941	-3,3327	-3,5394	-0,225	-0,2574	-0,2865
0	-4,3732	-3,7276	-3,0281	0,6269	0,6466	0,6741
1	-3,1164	-3,357	-3,5626	0,1538	0,1611	0,1553
0	3,545	3,6512	3,7218	0,8001	0,7883	0,7644
1	2,1731	2,2439	2,3093	0,0518	-0,0161	-0,0694
0	5,3087	5,2363	5,107	0,4943	0,3838	0,2728
1	3,778	3,8011	3,8118	0,3504	0,2806	0,2047
0	-1,3025	-1,4825	-1,6548	-0,1177	-0,1252	-0,1228
1	-1,2668	-1,576	-1,8659	-0,1431	-0,134	-0,1166
0	-0,7405	-0,9591	-1,1679	-0,1027	-0,0832	-0,0641
1	-1,5559	-1,9028	-2,2242	0,0636	0,1488	0,2349
0	0,2782	0,2581	0,2348	-0,12	-0,1182	-0,1166
1	0,2603	0,2636	0,266	0,0454	0,0459	0,0449
0	0,1797	0,1158	0,0494	-0,0877	-0,0892	-0,0888
1	0,348	0,3615	0,3744	0,0927	0,0897	0,0853
0	-1,6496	-1,8583	-2,0549	-0,5489	-0,5219	-0,489
1	-1,2742	-1,3667	-1,4604	-0,0434	-0,0715	-0,0948
0	-2,2814	-2,5787	-2,8516	-0,843	-0,7836	-0,6983
1	-1,1359	-1,2912	-1,4537	-0,1857	-0,2144	-0,2304
0	3,1131	3,3093	3,4901	1,3452	1,3376	1,327
1	2,8017	3,2324	3,612	0,9311	0,9549	0,946
0	3,3139	3,599	3,8695	1,1946	1,2522	1,3109
1	1,8985	2,3023	2,6551	1,3652	1,4391	1,5039
0	-2,6798	-2,7871	-2,8925	-0,183	-0,185	-0,1838
1	-3,1904	-3,6658	-4,0869	0,0047	0,0759	0,1535
0	-1,8589	-1,9212	-1,9827	-0,1081	-0,108	-0,1086
1	-2,4804	-2,8913	-3,2651	0,0309	0,1284	0,2377
0	3,5675	3,7279	3,8802	0,1252	0,0996	0,0566
1	4,6281	4,971	5,2891	0,235	0,1781	0,136
0	3,2675	3,3838	3,4866	-0,1497	-0,185	-0,2293
1	3,5571	4,0278	4,478	0,4258	0,4048	0,3974
0	-3,6318	-3,8468	-4,009	-0,4194	-0,3516	-0,2987
1	-1,5026	-1,7929	-2,0694	-0,9557	-0,8254	-0,6665
0	-3,7856	-3,9479	-4,0732	-0,3931	-0,32	-0,2575
1	-2,0567	-2,4673	-2,8325	-1,6467	-1,518	-1,3534
0	3,2105	3,1934	3,1625	0,6037	0,5918	0,5735
1	1,0468	1,2503	1,4645	0,2522	0,1923	0,1299
0	2,493	2,4526	2,3957	0,4839	0,5147	0,5378
1	0,7864	0,8805	0,9794	0,2587	0,2542	0,2437
0	-1,2804	-1,4031	-1,5232	0,026	-0,0089	-0,0376
1	-1,964	-2,0327	-2,0968	0,0201	0,015	0,0088
0	-1,3798	-1,5709	-1,7667	0,1226	0,0881	0,0477
1	-1,6103	-1,6669	-1,7202	-0,0436	-0,0596	-0,0771
0	2,4346	2,6725	2,8908	0,4555	0,3453	0,2051

1	0,6124	0,747	0,8812	-0,0773	-0,0981	-0,1192
0	0,9218	1,0242	1,1231	0,1798	0,1073	0,0251
1	1,3929	1,5693	1,7309	-0,0433	-0,0269	-0,0267
0	-0,9494	-1,0931	-1,2307	-0,155	-0,1281	-0,1007
1	-1,8353	-1,9394	-2,0344	-0,1505	-0,128	-0,1026
0	-1,409	-1,5987	-1,7786	-0,1138	-0,0674	-0,0295
1	-1,9414	-2,0518	-2,1496	-0,0898	-0,0821	-0,0801
0	1,1224	1,3245	1,5186	0,4423	0,3398	0,245
1	1,126	1,1262	1,1069	0,0923	0,1234	0,1384
0	0,9933	1,081	1,164	0,2545	0,2204	0,1893
1	1,2488	1,3094	1,3667	0,1348	0,0713	0,0037
0	-0,6058	-0,8427	-1,0862	-0,3218	-0,3187	-0,3189
1	-1,7205	-2,2856	-2,8346	-0,2552	-0,2448	-0,207
0	-1,24	-1,6868	-2,1257	-0,4014	-0,3803	-0,3369
1	-1,4314	-1,9859	-2,5379	-0,2893	-0,2696	-0,2256
0	0,8428	1,0388	1,2398	0,1417	0,1374	0,1396
1	4,2424	5,261	6,2508	0,0329	0,0664	0,1894
0	1,4142	1,7212	2,0327	0,236	0,2214	0,2165
1	3,3071	3,9903	4,6652	-0,0392	-0,0477	-0,0081
0	-2,0093	-2,6424	-3,2547	-0,1742	-0,1582	-0,0983
1	-2,042	-2,5661	-3,084	-0,6913	-0,4927	-0,2415
0	-2,7637	-3,2995	-3,8335	-0,2289	-0,2119	-0,1292
1	-3,8692	-4,6222	-5,3263	-0,5526	-0,2623	0,085
0	2,8991	3,5228	4,0958	-0,0208	-0,0189	-0,0101
1	3,6967	4,5408	5,3948	0,859	0,5837	0,3187
0	4,0897	5,107	6,0248	0,4496	0,5653	0,7349
1	4,6872	5,1727	5,6473	0,0624	-0,035	-0,1103

	Displacement (mm)		Strain (%)	
Torque (Nm/I	sup-mid	mid-deep	sup-mid	mid-deep
5	0	0,0019	0,0145	0,0113
5	0,2449	0,1236	0,0677	0,1015
6	0,0144	0,0121	0,0179	0,0145
5	0,5238	0,4997	0,0208	0,1188
40	0,0006	0,0007	0,0012	0,0014
48	0,0864	0,0643	0,0313	0,0352
55	0,0934	0,09	0,0932	0,0839
63	0,2532	0,2173	0,0866	0,072
7	0,1084	0,1191	0,0158	0,0183
6	1,0386	0,8975	0,6758	0,4679
6	0,1004	0,1094	0,0143	0,0103
6	0,8329	0,8026	0,0947	0,0886
35	0,066	0,0672	0,0072	0,0087
46	0,1141	0,1043	0,0004	0,0179
36	0,0584	0,0495	0,0026	0,0076
53	0,0139	0,0151	0,0014	0,0021
8	0,6411	0,6787	0,1774	0,2303
5	0,5121	0,536	0,0196	0,0284
8	0,8148	0,8696	0,0902	0,169
5	0,3195	0,3448	0,0006	0,017
32	0,3132	0,3219	0,1169	0,099
45	0,1953	0,2104	0,0352	0,0337
27	0,1933	0,183	0,1068	0,0847
39	0,137	0,1347	0,0549	0,0447
9	0,3403	0,3284	0,0163	0,027
5	0,1233	0,0944	0,1346	0,1417
6	0,3673	0,3538	0,0032	0,0065
5	0,0515	0,0947	0,1386	0,1595
70	0,2031	0,1697	0,1124	0,1094
51	0,0889	0,0924	0,0009	0,0031
24	0,0392	0,0333	0,0639	0,0575
47	0,0926	0,1	0,0075	0,0148
7	0,1332	0,1244	0,0276	0,0411
6	0,6142	0,5898	0,118	0,142
6	0,2138	0,1947	0,042	0,0153
6	0,5776	0,5538	0,0805	0,1078
95	0,1623	0,1364	0,056	0,0486
94	0,5215	0,4849	0,0319	0,0366
95	0,5601	0,5534	0,1079	0,0845
100	0,7528	0,7114	0,0061	0,0458
6	0,272	0,289	0,0802	0,074
6	0,6289	0,6419	0,188	0,247
6	0,2724	0,3144	0,0763	0,0727

7	0,423	0,4349	0,1361	0,1651
92	0,203	0,2073	0,0024	0,0264
105	0,3835	0,3735	0,0681	0,0711
111	0,2265	0,225	0,0182	0,0117
102	0,1244	0,1287	0,003	0,0013
11	0,0595	0,0884	0,0256	0,0135
6	0,3388	0,3485	0,0327	0,0596
9	0,0058	0,0277	0,0876	0,0604
6	0,32	0,3235	0,0355	0,0612
98	0,1601	0,169	0,0546	0,0515
91	0,2693	0,2782	0,0415	0,0424
110	0,2275	0,2324	0,091	0,0773
100	0,3951	0,4188	0,002	0,0245
10	0,1322	0,1336	0,0772	0,0723
10	0,3511	0,3647	0,0401	0,0637
10	0,1162	0,116	0,0757	0,0691
10	0,4189	0,4298	0,0926	0,1364
103	0,1291	0,1362	0,2586	0,2596
104	0,3021	0,3412	0,0623	0,0815
102	0,005	0,0072	0,0169	0,0156
110	0,194	0,1855	0,0323	0,0436
6	0,3508	0,3407	0,0027	0,0136
6	0,6795	0,6651	0,0054	0,0622
5	0,6066	0,586	0,2326	0,2614
7	0,8332	0,8194	0,0515	0,0236
24	0,0787	0,0803	0,0097	0,0029
28	0,1937	0,1931	0,1511	0,1547
23	0,2316	0,2522	0,0079	0,0248
31	0,1994	0,2024	0,0665	0,0761
7	0,7816	0,7953	0,2124	0,1062
6	0,4667	0,4776	0,0092	0,0289
6	0,7753	0,7634	0,1414	0,0719
8	0,44	0,4257	0,0018	0,0138
25	0,1486	0,1464	0,0133	0,0128
33	0,9437	0,9036	0,0397	0,0299
26	0,3044	0,2866	0,0173	0,0029
29	0,7174	0,6735	0,0655	0,005
5	0,1379	0,1373	0,0182	0,0103
5	0,4299	0,4134	0,0307	0,0512
5	0,0883	0,0886	0,0773	0,0594
5	0,3235	0,3142	0,0239	0,0405
62	0,4569	0,4695	0,2895	0,2728
89	0,019	0,0137	0,004	0,0065
50	0,3825	0,3643	0,0104	0,009
102	0,2364	0,2248	0,076	0,0823

5	0,3192	0,3448	0,0525	0,0521
5	0,2386	0,2067	0,0324	0,0291
5	0,6456	0,6995	0,0197	0,0275
5	0,2406	0,2056	0,0073	0,0058
64	0,1062	0,0706	0,0118	0,0239
59	0,0708	0,0654	0,0679	0,0533
92	0,0724	0,1293	0,1105	0,111
97	0,0231	0,0107	0,0698	0,0759
5	0,18	0,1723	0,0075	0,0024
6	0,3092	0,2899	0,0091	0,0174
6	0,2186	0,2088	0,0195	0,0191
6	0,3469	0,3214	0,0852	0,0861
73	0,0201	0,0233	0,0018	0,0016
92	0,0033	0,0024	0,0005	0,001
90	0,0639	0,0664	0,0015	0,0004
100	0,0135	0,0129	0,003	0,0044
6	0,2087	0,1966	0,027	0,0329
5	0,0925	0,0937	0,0281	0,0233
6	0,2973	0,2729	0,0594	0,0853
5	0,1553	0,1625	0,0287	0,016
95	0,1962	0,1808	0,0076	0,0106
100	0,4307	0,3796	0,0238	0,0089
91	0,2851	0,2705	0,0576	0,0587
83	0,4038	0,3528	0,0739	0,0648
18	0,1073	0,1054	0,002	0,0012
13	0,4754	0,4211	0,0712	0,0776
19	0,0623	0,0615	0,0001	0,0006
13	0,4109	0,3738	0,0975	0,1093
114	0,1604	0,1523	0,0256	0,043
115	0,3429	0,3181	0,0569	0,0421
120	0,1163	0,1028	0,0353	0,0443
109	0,4707	0,4502	0,021	0,0074
11	0,215	0,1622	0,0678	0,0529
15	0,2903	0,2765	0,1303	0,1589
12	0,1623	0,1253	0,0731	0,0625
15	0,4106	0,3652	0,1287	0,1646
109	0,0171	0,0309	0,0119	0,0183
105	0,2035	0,2142	0,0599	0,0624
91	0,0404	0,0569	0,0308	0,0231
115	0,0941	0,0989	0,0045	0,0105
4	0,1227	0,1201	0,0349	0,0287
5	0,0687	0,0641	0,0051	0,0062
4	0,1911	0,1958	0,0345	0,0404
5	0,0566	0,0533	0,016	0,0175
76	0,2379	0,2183	0,1102	0,1402

69	0,1346	0,1342	0,0208	0,0211
74	0,1024	0,0989	0,0725	0,0822
63	0,1764	0,1616	0,0164	0,0002
4	0,1437	0,1376	0,0269	0,0274
5	0,1041	0,095	0,0225	0,0254
4	0,1897	0,1799	0,0464	0,0379
5	0,1104	0,0978	0,0077	0,002
54	0,2021	0,1941	0,1025	0,0948
76	0,0002	0,0193	0,0311	0,015
97	0,0877	0,083	0,0341	0,0311
66	0,0606	0,0573	0,0635	0,0676
9	0,2369	0,2435	0,0031	0,0002
14	0,5651	0,549	0,0104	0,0378
10	0,4468	0,4389	0,0211	0,0434
13	0,5545	0,552	0,0197	0,044
111	0,196	0,201	0,0043	0,0022
130	1,0186	0,9898	0,0335	0,123
104	0,307	0,3115	0,0146	0,0049
128	0,6832	0,6749	0,0085	0,0396
12	0,6331	0,6123	0,016	0,0599
11	0,5241	0,5179	0,1986	0,2512
11	0,5358	0,534	0,017	0,0827
13	0,753	0,7041	0,2903	0,3473
108	0,6237	0,573	0,0019	0,0088
143	0,8441	0,854	0,2753	0,265
139	1,0173	0,9178	0,1157	0,1696
132	0,4855	0,4746	0,0974	0,0753
